# Discovering Causal Relationships in Grapevine Expression Data to Expand Gene Networks. A Case Study: Four Networks Related to Climate Change

**DOI:** 10.3389/fpls.2018.01385

**Published:** 2018-09-21

**Authors:** Giulia Malacarne, Stefania Pilati, Samuel Valentini, Francesco Asnicar, Marco Moretto, Paolo Sonego, Luca Masera, Valter Cavecchia, Enrico Blanzieri, Claudio Moser

**Affiliations:** ^1^Department of Genomics and Biology of Fruit Crops, Research and Innovation Centre, Fondazione Edmund Mach, San Michele all′Adige, Italy; ^2^Unit of Computational Biology, Research and Innovation Centre, Fondazione Edmund Mach, San Michele all′Adige, Italy; ^3^Department of Information Engineering and Computer Science, University of Trento, Trento, Italy; ^4^Consiglio Nazionale delle Ricerche-Institute of Materials for Electronics and Magnetism, Trento, Italy

**Keywords:** gene network, NES^2^RA, *Vitis vinifera*, climate change, abscisic acid ABA, flavonoids, stilbenoids, ERF

## Abstract

In recent years the scientific community has been heavily engaged in studying the grapevine response to climate change. Final goal is the identification of key genetic traits to be used in grapevine breeding and the setting of agronomic practices to improve climatic resilience. The increasing availability of transcriptomic studies, describing gene expression in many tissues and developmental, or treatment conditions, have allowed the implementation of gene expression compendia, which enclose a huge amount of information. The mining of transcriptomic data represents an effective approach to expand a known local gene network (LGN) by finding new related genes. We recently published a pipeline based on the iterative application of the PC-algorithm, named NES^2^RA, to expand gene networks in *Escherichia coli* and *Arabidopsis thaliana.* Here, we propose the application of this method to the grapevine transcriptomic compendium Vespucci, in order to expand four LGNs related to the grapevine response to climate change. Two networks are related to the secondary metabolic pathways for anthocyanin and stilbenoid synthesis, involved in the response to solar radiation, whereas the other two are signaling networks, related to the hormones abscisic acid and ethylene, possibly involved in the regulation of cell water balance and cuticle transpiration. The expansion networks produced by NES^2^RA algorithm have been evaluated by comparison with experimental data and biological knowledge on the identified genes showing fairly good consistency of the results. In addition, the algorithm was effective in retaining only the most significant interactions among the genes providing a useful framework for experimental validation. The application of the NES^2^RA to *Vitis vinifera* expression data by means of the BOINC-based implementation is available upon request (valter.cavecchia@cnr.it).

## Introduction

Climate change represents a major challenge for modern and future agriculture, in particular for grapevine cultivation and wine production due its wide geographical distribution, and economic relevance. A significant warming has already been observed in most grape-growing areas of the world affecting the varietal choice as well as grape quality (Mozell and Thach, 2014). The constant temperature increase trend has been triggering an advanced phenology, anticipating the ripening phase to warmer periods and affecting grape composition and development (Teixeira et al., 2013). An increased intensity of visible and UV solar radiation is also severely impacting on leaf morphological and biochemical parameters such as area, pigment, and antioxidant composition (Castagna et al., 2017), and berry characteristics, such as sugar-acid ratio and polyphenol abundance and composition (Bobeica et al., 2015). Finally, water deficit will be a major concern, not only in hotspots already facing seasonal drought, but also where rare heavy rains will alternate with drought during the same season. The scientific community is thus deploying great efforts to study the grapevine response to environmental changes (extensively reviewed in Geròs et al., 2015), with the aim to improve disease and yield prediction models and to identify key genetic traits to be used in grapevine breeding. Transcriptomic studies could be very useful to investigate plant adaptation as tuning of gene expression represents a major adaptive response of the plant. The increasing availability of grapevine transcriptomic data has prompted the creation of *Vitis*-gene expression compendia, such as VTCdb which included only microarray data (Wong et al., 2013) and Vespucci which comprises both microarray and RNA-seq data (Moretto et al., 2016, see **Table 1**). These databases contain a considerable amount of information that requires proper tools to be explored and interpreted. NES^2^RA algorithm (Asnicar et al., 2016) has been developed to expand known LGNs in model organisms, such as *E. coli* and *A. thaliana*, from transcriptomic data. It showed better performance than other approaches such as ARACNE (Margolin et al., 2006) and NESRA (Asnicar et al., 2015).

**Table 1:** Results of the expansion of the four grapevine gene networks using NES^2^RA.

Network name	Input genes	Genes/Interactions				Aggregated genes (*K* = 1000)
		*t* = 500,	*t* = 1000,	*t* = 500,	*t* = 1000,	
		*i* = 1000	*i* = 1000	*i* = 2000	*i* = 2000	
Anthocyanins	3	5962/6456	3570/3759	6485/7103	3944/4185	1056
Stilbenoids	13	15391/23459	10517/14443	16317/25666	11304/15904	1318
ERF/Ethylene	3	5817/6688	3516/3975	6337/7358	3911/4447	1146
ABA	5	7855/9562	4964/5941	8462/10425	5381/6504	1123

*t = tile size, *i* = iterations number*.

In this work we present the application of NES^2^RA to expand four grapevine LGNs related to the response to climate changes using the Vespucci compendium. To cope with the large computational requirement, NES^2^RA was run on the gene@home project developed on the Berkeley Open Infrastructure for Network Computing (BOINC) platform TN-Grid, which manages the distribution of the calculation among computers made available by the volunteers participating in the gene@home project (Asnicar et al., 2015). Here, we show that this approach can provide reliable results also in a non-model species such as *V. vinifera*, for which a much more limited amount of data is available. NES^2^RA final output consists in an expansion gene list and in an interaction file – defining the relationships among the genes – which are used to reconstruct the expansion network. This represents a helpful tool for biologists to expand LGNs based on *a priori* knowledge and/or experimental evidence on a system biology level providing novel candidates for further characterization. We focused on four grapevine networks possibly involved in the climate change response. Two networks are related to the secondary metabolic pathways for anthocyanin and stilbenoid synthesis, involved in the response to solar radiation, the remaining two are signaling networks, related to the hormones abscisic acid (ABA) and ethylene, involved in the regulation of cell water balance and cuticle transpiration. The network named “Anthocyanins,” i.e., the red pigments of grapes, comprises *VvUFGT*, which codes for the main enzyme responsible for anthocyanin biosynthesis (Ford et al., 1998), a cluster of four *VvMYBA* genes (Kobayashi et al., 2002; Walker et al., 2007) and *VvMYC1*, coding for transcription factors known to physically interact and regulate *VvUFGT* expression (Hichri et al., 2010). The network named “Stilbenoids,” i.e., grapevine secondary metabolites with plant defense properties (Mattivi et al., 2011), is composed by 13 genes: four *VvSTS* genes encoding for different stilbene synthase isoforms, the enzymes catalyzing the synthesis of resveratrol, *VvMYB14* and *VvMYB15*, coding for two characterized regulators of stilbene biosynthesis in grapevine (Höll et al., 2013) and seven genes encoding for peroxidases, enzymes potentially involved in the oligomerization of stilbene monomers. These enzymes have been included even if they have not been proved yet to be part of the stilbenoids regulatory network. The network named “ERFs” is formed by three genes coding for ethylene responsive transcription factors: *VvERF045* is up-regulated during berry ripening, while *VvERF042* and *VvERF044* are down-regulated and belong to the *SHINE* clade of AP2/ERF transcription factors that in *Arabidopsis* have been associated to cuticle structure, leaf and flower composition, and water loss (Aharoni et al., 2004; Shi et al., 2011; Leida et al., 2016). Finally, the network named “ABA” consists of five genes related to ABA signaling: three encoding for transcription factors, the ABA responsive element binding factor *AREB2*, the homeobox *HB7*, and *VvNAC26*; one for an ABA receptor homologous to the *Arabidopsis* ABA insensitive *ABI1* gene, and one for a receptor kinase *LRK10*. These genes are up-regulated by ABA treatment in grapevine cell cultures and pre-véraison berry skin samples (Nicolas et al., 2014; Pilati et al., 2017). *VvNAC26* expression affects berry size, likely by positively regulating ABA biosynthesis (Tello et al., 2015); *HB7* and *ABI1* are strictly interdependent in the ABA response in *Arabidopsis* (Soderman et al., 1996), whereas *LRK10* is related to ABA signaling and drought resistance in *Arabidopsis* (Lim et al., 2015).

## Materials and Methods

### Data Pre-processing of Vespucci Compendium

The Vespucci grapevine transcriptomic compendium (Moretto et al., 2016) available at http://vitis.colombos.fmach.it/, is composed by 29,090 genes and 1,565 contrasts, which refer to the comparisons of condition vs. reference within each experiment. The pre-processing procedure comprised three steps: (1) removal of contrasts with more than 55% of missing values; (2) removal of genes with more than 55% of missing values; (3) for each gene, replacement of the remaining missing values with the median of its contrasts values. This procedure reduced the original dataset to 28,013 genes and 701 contrasts (**Supplementary Table S1**).

### Gene Network Expansion by NES^2^RA Algorithm Within *gene@home* Project

The expansion lists were computed applying the NES^2^RA algorithm (Asnicar et al., 2016) to the pre-processed Vespucci dataset. NES^2^RA is based on the PC-algorithm, named after its authors Peter and Clark (Spirtes and Glymour, 1991), a gaussian graphical model (GGM) that finds causal relationships from observational data. Other GGM have been successfully applied to reconstruct plant regulatory gene networks (Ma et al., 2007), but the PC-algorithm has been shown to be the most promising for biological applications compared to Neighborhood selection, G-Lasso, and Shrinkage estimation (Albieri and Didelez, 2014).

**FIGURE 1:**
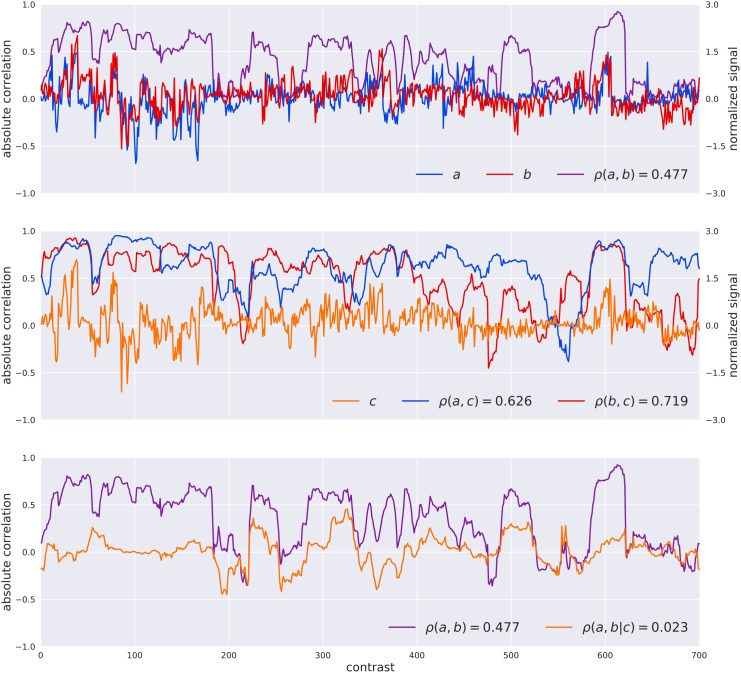
Visualization of the difference between the Pearson correlation [ρ(A,B)] of two genes A and B and their partial correlation given the gene C [ρ(A,B| C)] as computed by the PC-algorithm. The top plot reports the normalized raw signals of A and B, their windowed Pearson correlation (window-size of 31 experiments for visualization sake), and the value of their Pearson correlation coefficient in the legend. In the middle plot the signal of C is introduced as well as the windowed correlations between A,C and B,C and the values of correlation. The bottom plot shows the simple correlation between A,B and the partial correlation of A,B given C. In this case the PC-algorithm, that systematically search for variables for separating pair of variables, would have considered the correlation between A and B as completely explained in terms of C (a separation set of dimension one) and removed the interaction between them for the successive steps.

The PC-algorithm is based on a systematic test for conditional independence to retain significant relations between pairs of genes. It starts from a fully connected network and removes interactions between genes, if it finds a set of genes that supports that interaction (i.e., separation set). From a mathematical point of view, the test for statistical independence between the genes _a,b_ conditioned by a set of genes _S_ is driven by the estimation of the partial correlation _ρ(a,b∖S)_. The example reported in **Figure 1** shows how the estimated partial-correlation allows the identification of spurious interactions, with respect to the simple correlation. On the other hand, the exhaustive exploration of all the subsets of conditioning genes is computational impossible, therefore the PC-algorithm takes into consideration only a limited number of those sets, as described in Algorithm 3 in Asnicar et al. (2016). At the same time, the NES^2^RA algorithm, developed to cope with this computational complexity, randomly divides the Vespucci dataset into *tiles* of equal number of genes (subsetting), where each tile always includes all the genes of the LGN, to be then processed by the PC-algorithm. The random subsettings of all the genes in the genome are repeated for a given numbers of *iterations.* The NES^2^RA algorithm currently runs as part of the *gene@home* project which aims to systematically expand gene regulatory networks based on available public gene expression data. The *gene@home* project relies on thousands of volunteers’ computers by means of the BOINC system (Anderson, 2004) within the TN-Grid platform (Asnicar et al., 2015).

The networks found by the PC-algorithm are then post-processed off-line to determine and aggregate the final expansion gene lists. List aggregation is done with the ranking aggregation method Markov Chain 4 (MC4) (Dwork et al., 2001), since it has been shown to yield the most precise results (Asnicar et al., 2016). For the MC4 ranking aggregation we decided to consider the first 1,000 genes of each expansion output list (*K* = 1,000).

### Gene Network Expansion by Simple Correlation

To better show the difference between NES^2^RA and simple correlation results, the input networks were expanded computing the Pearson correlation coefficient between each input gene and all the other genes in the dataset, in pairs. The mean correlation of each gene respect to all the input genes was computed and sorted to provide the final expansion list.

### Networks Visualization

For each expanded gene network a limited number of genes were graphically depicted using Cytoscape (Shannon et al., 2003). In the case of the NES^2^RA networks, the putative interactions were determined using the interaction files produced by NES^2^RA as an intermediate output.

In the case of the simple correlation networks, an interaction between two genes was depicted if the Pearson coefficient passed the *Z*-test for correlation (*P*-value < 0.05).

### Validation Methods

Output genes were annotated according to the 12X PN40024 reference genome version 1 (V1) gene prediction annotation (**Supplementary Table S2** in Grimplet et al., 2012 and available at http://genomes.cribi.unipd.it/DATA/V1/ANNOTATION/), except for few transcription factor gene families recently characterized in grapevine, for which we referred to the specific published annotations (Ford et al., 1998; Gomez et al., 2009, 2011; Hichri et al., 2010; Licausi et al., 2010; Vannozzi et al., 2012; Wang et al., 2013, 2014; Liu et al., 2014; Cavallini et al., 2015; Wong et al., 2016). Two of the four networks produced by NES^2^RA were validated with published experimental datasets by computing a Precision curve to estimate the overlap between the two gene sets. Precision has been calculated as the intersection of the first k genes of the expansion lists calculated by NES^2^RA with the set of published experimental data, normalized on k. Additionally, two statistical approaches were applied to all the four networks: (i) the Gene Ontology functional category enrichment analysis, using the topGO Bioconductor package (Alexa and Rahnenfuehrer, 2015) coupled with the goslim_plant.obo available at http://www.geneontology.org, and (ii) motif enrichment promoter analysis using DREME (MEME suite, Bailey et al., 2009) and DAP motif database (O’Malley et al., 2016).

### Code Availability

A stand-alone version of the NES^2^RA pipeline is publicly available at https://github.com/lucamasera/NESSRA. In addition to the code, the repository contains the raw results presented in this paper before the aggregation step and the input files (local gene networks and the expression dataset) required to reproduce them. Note that, due to the random nature of the subsetting step, the results of the stand-alone version may differ from the ones computed by *gene@home* project presented in this work.

## Results

### Expansion of Four Grapevine Gene Networks

**FIGURE 2:**
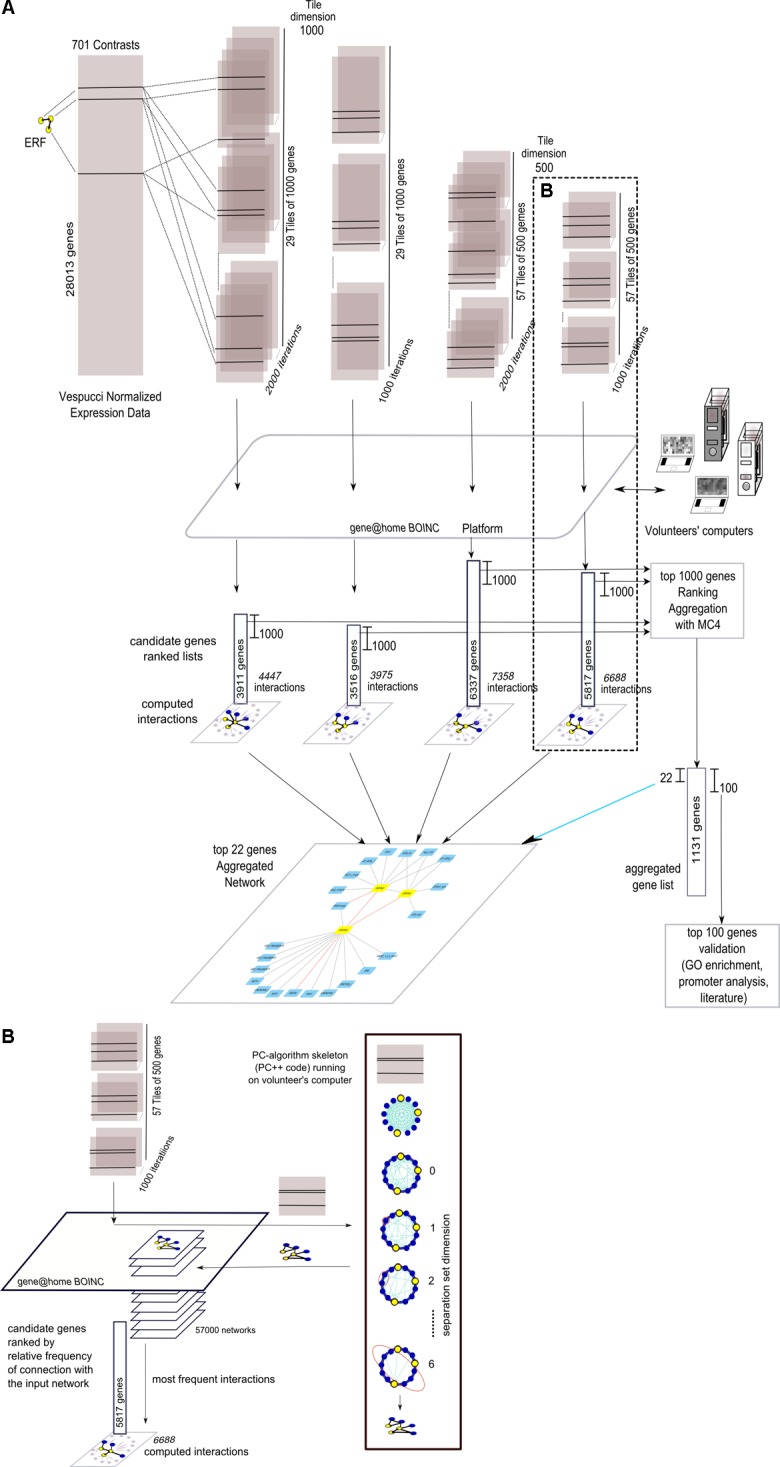
Overall view of the network expansion method. **(A)** Summary graphical visualization of the application (using four parameters settings) of NES^2^RA to the ERFs LGN using data from the Vespucci compendium. Each tile, including all the genes of the LGN, generated during the subsetting step, is analysed using the PC-algorithm that runs on the volunteers’ computers, made available through the *gene@home* BOINC project. The post-processing takes as input all the resulting networks and ranks the genes using their relative frequency, measured as the number of times a gene was found connected to the input LGN. In the final expansion network, the nodes are the LGN (in yellow) and the top-ranking genes of the aggregated list (in light blue), whereas the edges derive from the union of all the found interactions. **(B)** Details on the internal steps of the PC-algorithm that, starting from a complete graph, iteratively searches separation sets that allows the cut of an edge. For each tile, the result of the application of the PC-algorithm is a network.

The four grapevine gene networks, namely “Anthocyanins,” “Stilbenoids,” “ERFs,” and “ABA,” were expanded using NES^2^RA in order to identify additional genes causally related to them. The overall flow of analysis is depicted in **Figure 2A**, where the case of “ERFs” LGN is reported as an example. To reduce computing complexity, the Vespucci normalized expression dataset was divided into either 29 tiles of 1,000 gene (*t* = 1000) or 57 tiles of 500 genes (*t* = 500). This step was repeated 2,000 times (*i* = 2000) or 1,000 times (*i* = 1000). The three input genes of “ERFs” were present in each *tile*. The *gene@home* project allowed the distribution of the computation by the PC-algorithm as tiles to volunteers’ computers. Then, the partial results were post-processed to obtain four ranked lists of candidate genes and the corresponding interactions. The first 1,000 genes of each list were then aggregated using MC4 (*K* = 1000, see section “Material and Methods”). The first 22 genes of the aggregated list and their interactions with the “ERFs” network were used to depict the expanded network, whereas a larger list consisting of the top 100 genes was validated using Gene Ontology functional class enrichment, promoter analysis, and literature mining.

More in detail, as depicted in **Figure 2B**, given a *tile*, the PC-algorithm produced a network that represents the putative causal interactions between the genes. The PC-algorithm, starting from the complete correlation network and given a pair of interacting genes, searched for a growing-dimension separation set of genes within the genes interacting with the pair. The 57,000 networks thus obtained were post-processed, that is, interactions between genes, and “ERFs” genes were counted over the iterations and ranked accordingly. Finally, the candidate genes were ranked with respect to the overall relative frequency of interaction with the input network.

The results of the expansions of the four grapevine networks are presented in **Table 1**, where, for each set of parameters, the number of genes found and the number of interactions among them is reported. There is an evident dependence of the output gene number on the parameter settings. The first 1,000 genes of each of the four output lists were aggregated in order to have a refined final list, reported in the last column of **Table 1**.

### Expansion Networks Produced by NES^2^RA and Simple Correlation

**FIGURE 3:**
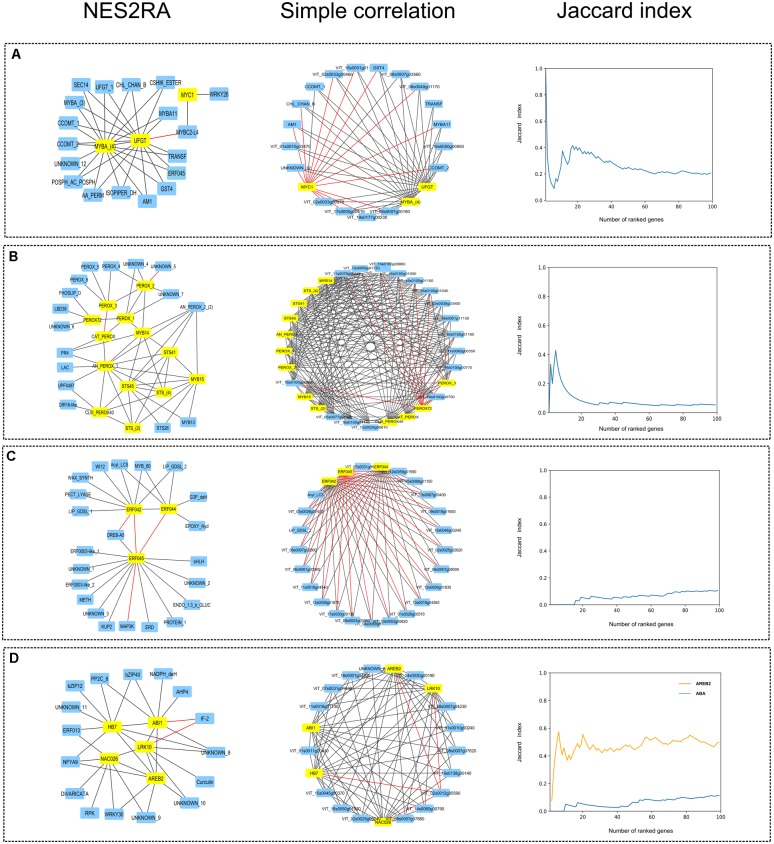
Comparison between expansion networks obtained with NES^2^RA and simple correlation. First column: visualization of the four gene networks obtained with NES^2^RA. Second column: visualization of the four gene networks obtained by computing simple correlation. Input genes have a yellow background, expansion genes a blue one. Gene names are abbreviations (see **Supplementary Table S4** for the complete description). Edges represent gene interactions: black and red lines link positively and negatively correlated genes, respectively. Third column: graphical representation of the Jaccard similarity index, calculated between the top-100 genes of the NES^2^RA and simple correlation expansion lists. **(A)** “Anthocyanins”, **(B)** “Stilbenoids,” **(C)** “ERFs,” and **(D)** “ABA” (and AREB2, in the Jaccard graph, orange line) networks.

In the attempt to highlight the difference between the expansion gene lists obtained using NES^2^RA and the simple correlation, both analyses have been performed on the same networks and the top 100 genes were considered for the comparison (**Supplementary Tables S2, S3**). One more network was computed, made of just one gene, the transcription factor AREB2 included in the ABA network, because it represents the simplest network possible. The networks produced by the two approaches have been visualized, considering only the top 20–30 genes of the lists for clarity (**Figure 3** and **Supplementary Table S4**). While in the case of the simple correlation networks the genes are almost fully connected, the number of interactions retained by NES^2^RA is considerably reduced. A clear effect of the latter approach is the removal of not significant interactions, possibly those due to noisy or redundant information. This allows to reduce the complexity of the network and focus on the network topology and the most likely gene interactions. Jaccard similarity index curves were calculated to compare the expansion gene lists obtained. Quite interestingly, the expansions of the single-gene network obtained with NES^2^RA and simple correlation resulted the most similar ones, with a rather constant high value of Jaccard index (corresponding to about 60% overlap). Conversely, the curves for the LGN expansions showed different trends and in general a lower similarity, suggesting that when a network rather than a single gene is expanded, the two approaches identify different sets of genes.

In the “Anthocyanins” network (**Figure 3A**), all the genes of the expansion except *VvMYBC2-L4* were positively correlated with both *VvMYBA* cluster (including *VvMYBA1* and *VvMYBA2*) and *VvUFGT*, highlighting the intimate relationship of this pair in regulating anthocyanin biosynthesis. *VvMYC1* was positively correlated exclusively with *VvWRKY26* and *VvMYBC2-L4*, which is in turn negatively correlated to *VvUFGT*. Concerning the “Stilbenoids” network (**Figure 3B**), characterized by a higher number of genes not fully confirmed by previous evidences, NES^2^RA analysis provided first of all an interesting analysis of the LGN, showing its lower homogeneity. Indeed, *VvMYB14* and *VvMYB15* were found interacting with all the stilbene synthases and three out of seven peroxidases, suggesting a minor involvement of the remaining four peroxidases in this pathway. Moreover, here we can appreciate the power of the PC-algorithm in eliminating spurious relationships in the case of *AN_PEROX, PEROX1*, and *VvMYB14*. Indeed, they are all highly co-expressed, but only the interactions between the two peroxidases and *VvMYB14* were retained. *VvMYB14* is indeed sufficient to explain the co-expression of the two enzymes, thus suggesting it could regulate the transcription of both. Widening to the expansion network, NES^2^RA identified one *STS* (*VvSTS26*), one *MYB* (*VvMYB13*) and a cluster of two peroxidase genes (*AN_PEROX_2*) related to the *VvMYB14-15* system and then other peroxidases and stress related genes connected to the other peroxidases. The topology of the “ERFs*”* expansion network (**Figure 3C**) revealed a bipartite structure with two *ERFs* anti-correlated to the third one and two groups of related genes, one enriched in transcription factors (lower group) and the other in genes related to lipid and wax metabolism (upper group). Finally, “ABA” network (**Figure 3D**) was characterized by a rather homogeneous distribution of the interactions both in the input and in the expanded gene sets. The interaction between *HB7_2* and *ABI1* was confirmed. In general, the three transcription factors were connected to many other regulators, including a Nuclear Factor Y, whereas the two kinases interacted with other signaling proteins (*DBPPP2C_6, AHP4*).

### Validation of the Expanded Gene Networks

To estimate the biological relevance of the genes identified by NES^2^RA, we searched for public experimental data – not included in Vespucci compendium – with an experimental design suited to validate our networks and we could find them only for “ABA” and “Anthocyanins” networks. The dataset for “ABA” consisted in the genes modulated by ABA treatment in grapevine berry skins and in cell cultures (Nicolas et al., 2014; Pilati et al., 2017); for the Anthocyanins network the dataset consisted in the differentially modulated genes in grapevine hairy roots overexpressing *VvMYBA1* (Cutanda-Perez et al., 2009). As shown in **Supplementary Figure S1** precision is initially very high and then decreases with the number of genes, suggesting that genes are ranked in a biologically relevant way. Considering the top 100 genes precision decreases to 40 and 20% in the case of “ABA” and “Anthocyanins,” respectively, suggesting that the top 100 genes of the expansion lists represents a reasonable trade-off between focusing on the strongest candidates of the expanded networks and preserving enough statistical power for the other analyses. Thus, this threshold was applied to the other two networks too.

The four gene expansion lists were analyzed with TopGO (Fisher’s exact test) to get a general overview of the more represented functional categories (**Supplementary Table S5**). Despite the high number of unknown or non-annotated genes, this analysis showed that the top 100 genes in the NES^2^RA output were functionally related to the input genes: the “Anthocyanins” and “Stilbenoids” expansions were populated by genes related to secondary metabolism and defense, while the “ERFs” and “ABA” expansions were populated by many regulators and genes involved in the lipid biosynthesis and in the hormonal and abiotic stress responses, respectively.

To verify if the expanded networks included co-regulated genes, sharing common regulatory motifs in their promoters, the 1 Kb promoter regions of the top 100-gene lists were retrieved and analyzed with DREME (Bailey et al., 2009). Results are presented in **Supplementary Table S6**.

Promoter analysis produced different outputs for the four networks. The promoters of the genes of the “Anthocyanins” network were enriched in two 8-nt motifs, which were not present in the reference annotation database (O’Malley et al., 2016), meaning that these sequences have not been already characterized and therefore cannot be associated yet to a transcription factor. “ABA” network was highly enriched in three 8-nt motifs well annotated in the reference databases: two of them (CACBTGTC and CCACGTGK) matched with the motifs recognized by bHLH and/or bZIP AREB transcription factors and one (ACVCTCCT) by MYB88. “Stilbenoids” network retrieved a sequence of just 5-nt (ACGYG), rather unspecific since matching to 72 possible transcription factors belonging to several transcription factor families. Finally, in the “ERFs” network no enriched motifs could be found. Overall, this analysis suggests that despite the starting networks were composed, partly or exclusively, by transcription factors, the expansion sets not necessarily are enriched in their direct targets. Further experiments would be necessary to come to a more general conclusion.

## Discussion

In the field of viticulture, growing attention is dedicated to investigate plant adaptation and stress responses to climate change, as this represents the main challenge for the near future (Geròs et al., 2015). Here we present an *in silico* approach to extend our knowledge on gene networks related to plant-environment interaction, relevant for berry quality and water stress response, with the final aim to identify novel candidates to drive experimental work. Anthocyanin and stilbenoid synthesis are known to be deeply affected by changes in external conditions, such as air temperature, water availability and solar radiation type and intensity (Teixeira et al., 2013). Besides, specific ethylene responsive factors are reported to be involved in cuticle metabolism thus regulating water transpiration (Aharoni et al., 2004; Shi et al., 2011) and ABA is intimately related to plant water balance and drought stress tolerance (Fujita et al., 2011; Golldack et al., 2014). Despite the role of these cellular processes and/or factors has been well ascertained at the physiological level, much remains to be elucidated at the molecular level.

Gene network computation based on large datasets of expression data represents an interesting approach to unveil the complex interactions between transcripts inside the cell. Here we present the application of a PC-based algorithm NES^2^RA (Asnicar et al., 2016) to the grapevine transcriptomic compendium Vespucci (Moretto et al., 2016) in order to expand LGNs (**Figure 2**). NES^2^RA was initially set up to analyze model organisms transcriptomic data, such as *E. coli* and *A. thaliana*, where a large number of experimental conditions are available. However, considering NES^2^RA ability to scale to lower data dimensions, we were interested in testing its performance on a non-model species such as *V. vinifera*. Compared to simple correlation approaches, NES^2^RA applies a statistical criterion of conditional independence which, starting from a fully connected network, eliminates spurious and/or redundant relationships (**Figure 1**). This simplifies the network and highlights significant relationships between genes, named interactions. The same challenge has been addressed in other works by calculating a co-expression index [based on highest reciprocal ranks (HRR) and mutual ranks (MR)] within the VTCdb (Wong et al., 2013) and *ad-hoc* datasets in specific studies, also including promoter *cis*-regulatory elements analysis (Wong et al., 2016, 2017; Vannozzi et al., 2018). While expanding a single gene produces similar results with all the approaches, the power of NES^2^RA becomes more evident when expanding LGNs (**Figure 3**). Moreover, the expansions obtained with NES^2^RA strictly depend on the nature of the LGN for two reasons: firstly, the input genes define the separation set during the PC-algorithm run, secondly, the post-processing pipeline top-ranks genes with the largest number of connections to the whole LGN. Therefore NES^2^RA preferentially identifies highly connected central nodes rather than one-to-one direct gene interactions, such as the case of a transcription factor and its target. The comparison of NES^2^RA expansions with experimental datasets found in literature confirmed that the top-ranking genes were modulated upon ABA stimulus or *VvMYBA1* over-expression supporting they belong to these networks (**Supplementary Figure S1**). The Gene Ontology enrichment highlighted for all the networks functional categories related to the LGNs (**Supplementary Table S5**). Conversely, promoter motif enrichment analysis produced more case-specific results (**Supplementary Table S6**): the “Anthocyanins” and “ABA” expansions genes were enriched in motifs, uncharacterized in the former case and recognized by ABA responsive transcription factors in the latter; “Stilbenoids” and “ERFs” expansions genes didn’t show any significant co-regulation, despite the fact that the second network included exclusively transcription factors. We can speculate that this result might be ascribed to the heterogeneity of the input network and/or to the amplification of the signaling cascade in the expansion network.

Going into the detail of each expanded network, the *VvMYBA* cluster (including *VvMYBA1* and *VvMYBA2*) was found to interact with *VvUFGT* and both genes were positively correlated with *VvAM1* and *VvGST4*, known to be involved in vacuolar sequestration of anthocyanins (Gomez et al., 2009, 2011). Interestingly, *VvMYBA* and *VvUFGT* were found interacting with *VvERF045*, supporting the recently suggested role of this factor in regulating genes of the anthocyanin pathway (Leida et al., 2016), and with other five *MYBA* regulators and enzymes of the general phenylpropanoid pathway (*CCOMT* and *CSHIK*_*ESTER*). The MYC1 interaction with the two transcription factors VvWRKY26 and VvMYBC2-L4, recently characterized as regulators of both Anthocyanin and PAs biosynthesis (Cavallini et al., 2015; Amato et al., 2017), confirms its involvement in both pathways. Apparently, NES^2^RA is not able to clearly solve the cases of multifactors-mediated regulation such as the case of the known MBW mode of action. However, we cannot appreciate whether this is due to the mining approach or to the intrinsic multilevel nature of the regulation which could be not purely transcriptional.

Considering the “Stilbenoids” expansion network, the already known interactions within the input gene set were confirmed: *VvMYB14* and *VvMYB15* were found to interact with eight stilbene synthases (*VvSTS25*/*27*/*29*/*31, VvSTS41, VvSTS45*, and *VvSTS35*/*48*) as supported by the recent evidences by Wong et al. (2016) and Vannozzi et al. (2018), and with two peroxidases, *AN_PEROX_1* and *CLIII_PEROX40*, both co-expressed with *VvSTS41* and *VvSTS45* in VTCdb database. In addition, NES^2^RA identified another MYB factor (*VvMYB13*), which shares close similarity in sequence and expression with *VvMYB14* and *VvMYB15* (Wong et al., 2016), and additionally a STS (*VvSTS26*) and a cluster of two peroxidases (*AN_PEROX_2*) which will be tested for their ability to catalyze stilbenoids synthesis and oligomerization during the grapevine response to abiotic and biotic stresses. The group of *PEROX_1, _2, _3*, interacting with Vv*MYB14*, is found related to other peroxidases (*PEROX_4, _5, _6*), which could participate to stilbenoid metabolisms, as well.

Interestingly, among the top-100 genes of the “Stilbenoids” LGN expansion (**Supplementary Table S2**), there is a Group II WRKY transcription factor, *VvWRKY18*, which represents a potential new interesting candidate for the regulation of this pathway. On the other hand, the *VvWRKY* genes proposed by Vannozzi et al. (2018) as involved in the regulation of *VvSTS* genes are not present in the NES^2^RA output. The approach of Gene Co-expression Network analysis based on the MR index of co-expression proved to be more suited to identify direct interactions, such as the regulator of a target. Nonetheless, NES^2^RA expansion of *VvSTS29* actually identifies *VvWRKY03* (not shown), confirming that when applied to a single gene, the two approaches produce similar results.

“ERFs” expansion network reflected the bipartite structure of the input set, in which the two SHINE genes (*VvERF042/44*) were inversely related to *VvERF045.* The only common gene is represented by a DREB transcription factor. *VvERF042/44*, uncharacterized in grapevine, were found correlated with numerous genes involved in cuticle metabolism such as *Acyl-LCS, WAX SYNTH, LIP-GDSL, EPOXY-Hyd* (Yeats and Rose, 2013) and one gene linked to stomatal closure regulation, *VvMYB60*, (Galbiati et al., 2011), supporting the hypothesis that also in grapevine they could play a role in the control of transpiration. Interestingly, *VvERF042* and *VvERF044* are the putative orthologs of the *Arabidopsis* *SHINE1* and *SHINE3* genes, whose silencing leads to a decrease in cutin load and to changes in cell wall structure (Shi et al., 2011), and they resulted down-regulated in grapevine transgenic lines overexpressing *VvERF045* (Leida et al., 2016). *VvERF045* which is specifically induced at véraison (Leida et al., 2016), seems to interact with other transcription factors (*ERF0003-like, bHlH*) and genes involved in signaling, suggesting it could be a higher level regulator of berry ripening. “ABA” input network showed a complete connection between *ABI1, HB7, AREB2* and *LRK10*, thus representing a very co-expressed module, *VvNAC026* instead was exclusively connected to *AREB2* and *LRK10* and could represent a second-order, more specialized, level of regulation. This interaction supports the involvement of *VvNAC026* in berry size determination via positive regulation of ABA synthesis, as recently proposed by Tello et al. (2015). The expanded network was highly enriched in signaling components, such as transcription factors (*VvbZIP12, VvbZIP40, VvWRKY30, Divaricata, VvERF13, NF-YA9*) and kinases/phosphorilases (*PP2C*_6, *AHP4, RPK*), thus proposing novel candidates to expand ABA regulatory network. Interestingly, the Nuclear Factor *YA9* is reported to be induced by salt and PEG treatments and possibly related to seed maturation and dehydration signaling in grapevine (Ren et al., 2016).

## Conclusion

In conclusion, we have shown that NES^2^RA algorithm can be successfully applied to a non-model plant species transcriptomic data to expand LGNs, not necessarily fully characterized. It is meant to be used on a complete database including RNA-seq and microarray data - such as Vespucci - without losing the ability of identifying local specialized networks, in this study the flavonoid-related LGNs. Indeed, it represents a useful tool to mine the entire transcriptome to extend a LGN, on a system biology perspective, as it searches for genes highly connected to the whole LGN. For instance, it seems to find points of convergence between pathways, such as the case of the *VvMYC1* gene bridging the Anthocyanin and Proanthocyanidin synthesis pathways. In the case of the ABA expansion, NES^2^RA identifies many genes known to be related to this signaling network and many other transcription factors and genes representing interesting candidates for experimental characterization. However, we observed that NES^2^RA does not represent the best tool to find direct targets of transcription factors unless the single gene is analyzed.

Since this approach is computationally demanding, we have overcome this problem by exploiting the BOINC platform, with the drawback of not providing a user-web resource. We are currently developing alternative strategies to solve this problem.

## Author Contributions

EB and CM conceived, developed, and coordinated the research. SP, GM, and CM identified the networks and analyzed the expansion results. MM and PS helped with the expression compendium. SV and FA contributed to the whole data analysis, to results aggregation and networks drawing. LM and VC assisted with the BOINC application. GM and SP contributed substantially to manuscript preparation. All authors read and approved the final version of the manuscript.

## Conflict of Interest Statement

The authors declare that the research was conducted in the absence of any commercial or financial relationships that could be construed as a potential conflict of interest.
