# Supplementary figures and images for: Discovering Causal Relationships in Grapevine Expression Data to Expand Gene Networks. A Case Study: Four Networks Related to Climate Change
Source: Front Plant Sci. 2018 Sep 21;9:1385. doi: 10.3389/fpls.2018.01385 (PMC6161569; doi:10.3389/fpls.2018.01385)

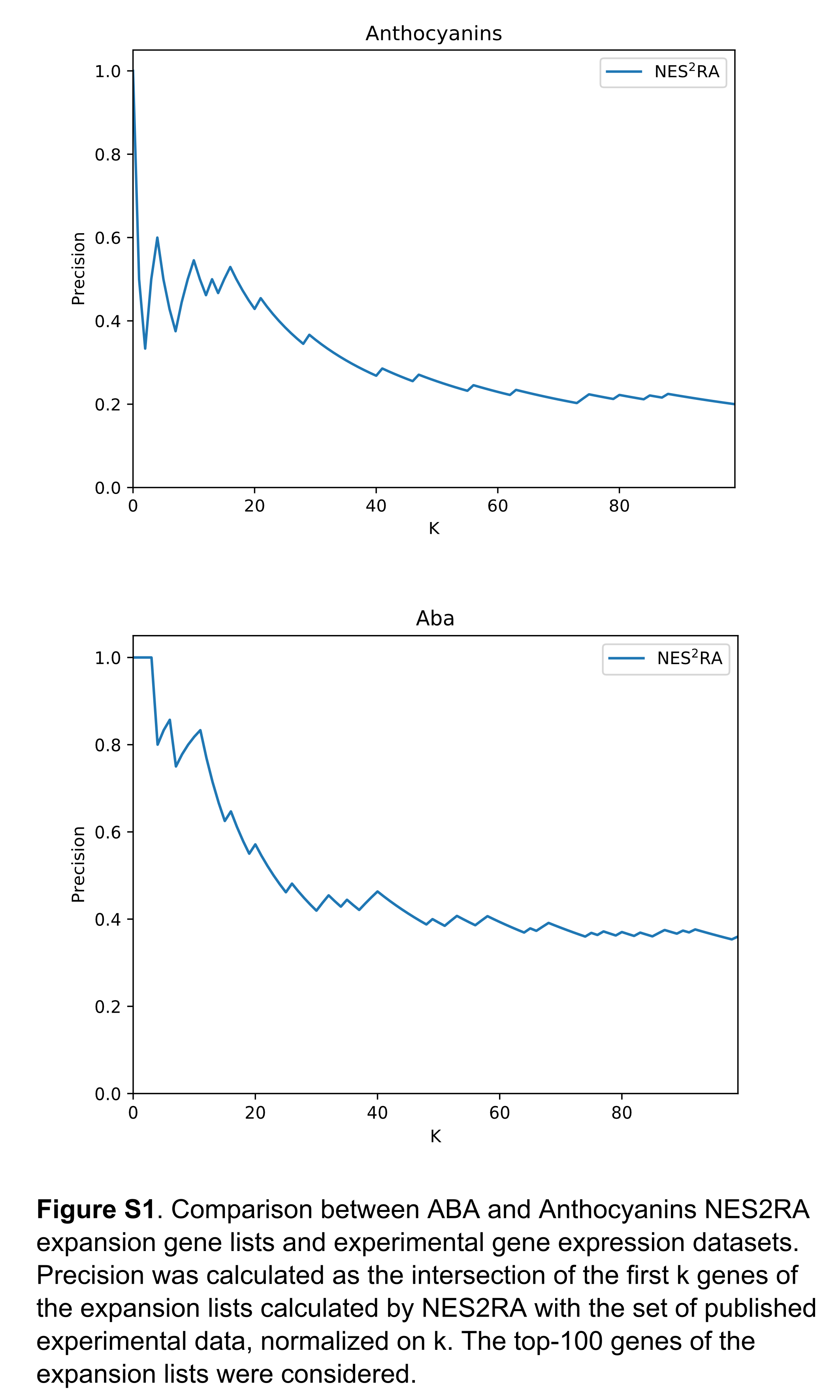

Supplement: Supplementary file 7 [file Image_1.TIF]
